# Inflammatory bowel diseases in Brazil: journey of doctors who care for patients. What is the importance?

**DOI:** 10.1590/0102-67202025000023e1892

**Published:** 2025-08-18

**Authors:** Marcela Maria Silvino CRAVEIRO, Lígia Yukie SASSAKI, Eduardo Garcia VILELA, Rogério Saad HOSSNE

**Affiliations:** 1 Universidade Estadual Paulista, Faculty of Medicine, Department of Surgery and Orthopedics – Botucatu (SP), Brazil.; 2 Universidade Estadual Paulista, Faculty of Medicine, Department of Clinical Medicine – Botucatu (SP), Brazil.; 3 Universidade Federal de Minas Gerais, Faculty of Medicine, Department of Clinical Medicine – Belo Horizonte (MG), Brazil.

**Keywords:** Health Services Accessibility, Health Policy, Inflammatory Bowel Diseases, Crohn’s Disease., Acessibilidade aos Serviços de Saúde, Política de Saúde, Doenças Inflamatórias Intestinais, Doença de Crohn

## Abstract

**Background:**

Inflammatory bowel diseases (IBDs) are chronic inflammatory conditions of a recurrent nature, whose incidence and prevalence rates have increased worldwide.

**Aims:**

The aim of this study was to profile the doctors who treat patients with IBDs in Brazil and to understand and analyze the journey and importance of this care.

**Methods:**

This is a cross-sectional study that descriptively and inferentially analyzed the pre-existing database of the Brazilian Inflammatory Bowel Disease Study Group and through this observed the reality of care for Crohn’s disease and ulcerative colitis, in the country.

**Results:**

In the descriptive analysis, we found results regarding the physicians’ profile such as specialty, number of patients treated with these diseases and their difficulties in accessing medications, complementary exams, and multidisciplinary team. In the statistical analysis regarding the Human Development Index of the states, the significant results were related to workplace, difficulty in accessing medications, and referral to other specialists. Regarding the association of variables with medical demographics, the results were significant in relation to workplace, difficulty in accessing medications, and complementary exams.

**Conclusions:**

The study showed a profile of the doctors who treat patients with IBDs and who participated in this survey. In addition to analyzing and describing the doctor’s profile and their difficulties, we listed the main aspects that hinder both diagnosis and treatment, attributed to external factors, regardless of their reality and competence.

## ARTICLE HIGHLIGHTS

- Inflammatory bowel diseases (IBDs), represented by Crohn’s disease and ulcerative colitis, are conditions whose epidemiological rates are increasing worldwide.
- The study of IBDs and the treatment of patients with these conditions are a daily challenge for specialist doctors.
- Understanding the profile of the doctors who treat these patients and their difficulties during treatment is essential.
- Many adversities are related to health policies, such as access to medications and complementary tests, which compromises the adequate treatment of these patients.

## CENTRAL MESSAGE

Inflammatory bowel diseases are chronic inflammatory conditions of a recurrent nature, whose incidence and prevalence rates have increased worldwide. It is known that early diagnosis and short start of the correct indicated treatment alter the natural history of the disease, preventing complications; hence, it is necessary to know the profile of the doctors who treat these patients in Brazil and especially to understand the difficulties in care and evaluate them in relation to other variables.

## PERSPECTIVES

This study showed the profile of physicians who treat inflammatory bowel disease (IBD) patients, through their registration in the Brazilian Inflammatory Bowel Disease Study Group. In addition to analyzing the physician’s profile and their difficulties, the main aspects that hinder both the diagnosis and treatment of the disease, attributed to external factors, were listed. Therefore, more effective public health policies should be planned and expanded, aiming at growth and adaptation focused on IBDs.

## VISUAL ABSTRACT

**Figure:**
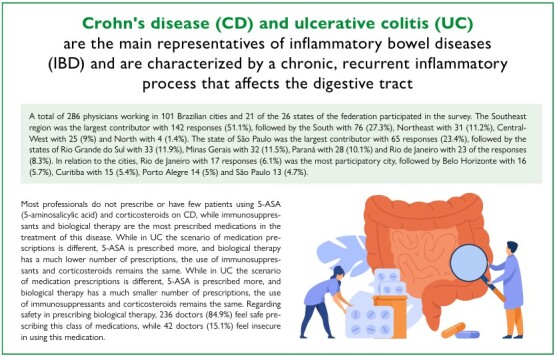

## RESUMO

**Racional:**

As doenças inflamatórias intestinais são afecções inflamatórias crônicas de caráter recorrente, cujas taxas de incidência e prevalência têm aumentado mundialmente.

**Objetivos:**

Traçar o perfil dos médicos que atendem pacientes com doenças inflamatórias intestinais no Brasil, compreender e analisar a jornada e importância deste atendimento.

**Métodos:**

Trata-se de um estudo transversal que analisou de forma descritiva e inferencial o banco de dados pré-existente do Grupo de Estudos da Doença Inflamatória Intestinal do Brasil, observando a realidade do atendimento da doença de Crohn e retocolite ulcerativa no país.

**Resultados:**

Na análise descritiva, encontramos resultados quanto ao perfil do médico como: especialidade, número de pacientes atendidos com estas doenças e suas dificuldades de acesso a medicamentos, exames complementares e equipe multidisciplinar. Na análise estatística referente ao Índice de Desenvolvimento Humano dos estados, os resultados significativos foram em relação: ao local de trabalho, dificuldade de acesso a medicamentos, encaminhamento para outros especialistas. Quanto à associação das variáveis com a demografia médica os resultados foram significativos em relação a: local de trabalho, dificuldade de acesso a medicamentos e exames complementares.

**Conclusões:**

O estudo mostrou um retrato do perfil dos médicos que atendem portadores de doenças inflamatórias intestinais e que participaram deste levantamento. Além da análise e descrição do perfil do médico e de suas dificuldades, relacionamos os principais aspectos que prejudicam tanto ao diagnóstico e o tratamento, atribuídos a fatores externos, independente da sua realidade e competência.

## INTRODUCTION

 Crohn’s disease (CD) and ulcerative colitis (UC) are the main representatives of inflammatory bowel diseases (IBDs) and are characterized by a chronic, recurrent inflammatory process that affects the digestive tract. Their etiology is still unknown; however, there have been advances in the knowledge of the pathophysiology, and it is known that these are diseases with a complex relationship between genetic, environmental, and immunological factors^12,15^. 

 Due to their chronicity, their evolution, the extent of their involvement in the digestive tract, and the progressive tissue damage, IBDs have a major impact on patients’ quality of life and high morbidity rates^4,6,7,13^. 

 These patients are diagnosed at increasingly younger ages, usually close to the age of entry into the job market, which is related to higher rates of absence from work and use of highcost medications for longer periods, resulting in higher costs and requiring more targeted and specific public health policies. In developing countries, this impact can be even more burdensome^9^. In this scenario, there is also an increase in incidence and prevalence rates in these countries, which, together with the aging of the population, contributes to the increase in costs related to the management of the disease and its complications, requiring more targeted and specific policies. Preventive measures, mainly related to healthier lifestyle habits, advances in research, and reformulation of social security may prove to be fundamental in minimizing costs and ensuring adequate treatment for patients with this condition^5,10^. 

 Therefore, the role and performance of physicians who treat patients with IBDs become crucial; however, data on the journey of these physicians are scarce in the world literature. In Brazil, due to its continental characteristics, regional diversity, unequal distribution of physicians and income, and difficulties in accessing treatment, among others, this scenario becomes even more challenging. This characterization, together with the profile of these physicians, can help in decision-making in health policies, training and strengthening the actions of civil societies and scientific societies. 

 In Brazil, we do not have data on the journey and profile of doctors who treat patients with IBDs nor on the resources, difficulties, location, and access to treatment in health systems. This study aimed to highlight these realities from the perspective of doctors who treat these diseases. 

## METHODS

 This is a cross-sectional study that used a pre-existing database from the Brazilian Inflammatory Bowel Disease Study Group (GEDIIB). The study was submitted to and approved by the Research Ethics Committee of the Botucatu School of Medicine (FMB/UNESP) (42079120.0.0000.5411). These data were previously obtained through a specific Google Forms questionnaire, sent in the form of a "Quiz" to the research participants. 

 Physicians who were known to be references for IBD care in their cities or macro-regions and/or members of GEDIIB were invited to participate in the research. Incomplete questionnaires and questionnaires completed by professionals working in pediatric gastroenterology were excluded since the focus of the study was only on the care of adult patients. Responses from the period between April and October 2019 were accepted. 

 The variables analyzed in the questionnaire were city and state where they work; time since graduation; medical specialty; years of experience in the specialty; workplace; difficulty in accessing and dispensing medications for patients with CD and UC; difficulty in referring to other specialists who work with IBD; difficulty in performing complementary exams; number of patients with CD and UC; number of patients with CD and UC using each class of medications; number of patients who use each class of biological therapy as first and second choices for approaching CD and UC; and which topics within the approach to IBD they would like to discuss. 

 In order to equalize the data and analyses based on the different local and regional aspects, the Human Development Index (HDI) was used, obtained through the website of the Brazilian Institute of Geography and Statistics (IBGE)^8^ which is stratified as follows: 0.800–1.000, very high; 0.700–0.799, high; 0.555–0.699, medium; and 0.350–0.554, low. 

 Another measure used to adjust the data and analyses was the medical ratio, obtained from a formula adopted by the Organization for Economic Cooperation and Development (OECD), an organization whose function is to present data and prepare reports on macroeconomics, trade, development, education, science, and innovation among the countries of Europe, Asia, Oceania, and the Americas, including Brazil. In this regard, the ratio is based on the number of doctors per 100,000 inhabitants and the reference value adopted is 3.5 doctors/1,000 inhabitants. For the study, states with a ratio equal to or greater than the value adopted by the OECD^11^ and lower than this reference were dichotomized. The data on the medical ratio for each state were extracted from the latest Medical Demography in Brazil^14^. 

 Statistical analyses were performed using the SPSS for Windows version 23.0 (SPSS Inc., Chicago, IL, USA). Numerical variables were assessed for normality using the Kolmogorov-Smirnov test to select the presentation of data, and categorical variables were presented as absolute values and percentages. Variables were compared using the Student’s ttest or Mann-Whitney test (according to the distribution of data) and chi-square (or Fisher’s exact test when appropriate). The p-value adopted was less than 0.05. 

## RESULTS

 A total of 286 physicians working in 101 Brazilian cities and 21 of the 26 states of the federation participated in the survey. 

 The Southeast region was the largest contributor with 142 responses (51.1%), followed by the South with 76 (27.3%), Northeast with 31 (11.2%), Central-West with 25 (9%), and North with 4 (1.4%). 

 The state of São Paulo was the largest contributor with 65 responses (23.4%), followed by the states of Rio Grande do Sul with 33 (11.9%), Minas Gerais with 32 (11.5%), Paraná with 28 (10.1%), and Rio de Janeiro with 23 of the responses (8.3%). In relation to the cities, Rio de Janeiro with 17 responses (6.1%) was the most participatory city, followed by Belo Horizonte with 16 (5.7%), Curitiba with 15 (5.4%), Porto Alegre with 14 (5%), and São Paulo with 13 (4.7%). 

 The median time since graduation of participants was 17 years (3–55 years), while the median time since specialization was 13 years (minimum range of 1 year and maximum of 51 years). The specialty that contributed the most was gastroenterology with 140 responses (50.4%), followed by coloproctology with 104 (37.4%), digestive system surgery with 13 (4.7%), digestive endoscopy with 11 (4.0%), general surgery with 7 (2.5%), and clinical medicine with 1 (0.4%). The majority of physicians work in private practices/clinics, adding up to 248 professionals (89.2%), 144 (51.8%) in public hospitals, 133 (47.8%) in private hospitals, 108 (38.8%) in public outpatient clinics, 73 (26.3%) in public outpatient clinics specialized in IBDs, and 23 (8.3%) in private practices/clinics specialized in IBDs. Among these professionals, 57 (20.5%) work in only one place, 67 (24.1%) in three places, 72 (25.9%) in more than three places, and 81 (29.1%) work in two places. Regarding access to and release of medications, for patients with CD, biological therapy was the drug class that generated the most difficulties in obtaining, with 194 responses (69.8%), followed by 6-mercaptopurine with 64 responses (23%), 5-aminosalicylic acid (5-ASA) derivatives with 50 responses (18%), methotrexate with 38 responses (13.7%), azathioprine with 37 responses (13.3%), and corticosteroid with 8 responses (2.9%). For the treatment of UC, biological therapy remained in first place with 264 responses (95%), followed by 6-mercaptopurine with 51 (18.3%), 5-ASA derivatives with 49 (17.6%), azathioprine with 38 (13.7%), and corticosteroid with 5 (1.8%). 

 In the interdisciplinarity item, referral to a nutrition professional was reported as the most difficult with 111 responses (39.9%), in second place psychology with 110 (39.6%), in third nursing with 66 (23.7%), in fourth pathology with 48 (17.3%), in fifth surgery with 43 (15.5%), and in sixth gastroenterology with 21 (7.6%). For the propaedeutic approach of patients, the greatest difficulty was access to double-balloon enteroscopy with 218 responses (78.4%), followed by capsule endoscopy with 212 (76.3%), fecal markers with 188 (67.6%), entero resonance with 141 (50.7%), enterotomography with 110 (39.6%), tuberculosis (TB) screening with 87 (31.3%), colonoscopy with 54 (19.4%), esophagogastroduodenoscopy (EGD) with 19 (6.8%), anatomopathological with 14 (5%), serum markers with 7 (2.5%), X-ray for tuberculosis screening (X-ray TB) with 6 (2.2%), and viral serology and laboratory routine with 2 responses (0.7%). For both CD and UC, most physicians reported seeing between 11 and 50 patients (40.6% and 41.4%, respectively). Most professionals do not prescribe or have few patients using 5-ASA derivatives and corticosteroids on CD, while immunosuppressants and biological therapy are the most prescribed medications in the treatment of this disease. While in UC the scenario of medication prescriptions is different, 5-ASA derivatives are prescribed more, and biological therapy has a much lower number of prescriptions, the use of immunosuppressants and corticosteroids remains the same. Regarding safety in prescribing biological therapy, 236 doctors (84.9%) feel safe prescribing this class of medications, while 42 doctors (15.1%) feel insecure about using this medication. Among the main reasons for insecurity related to the use of this therapy, the lack of discussion with more experienced teams and doctors stands out. 

 In the treatment of CD, anti-tumor necrosis factor (anti-TNF) drugs, adalimumab, and mainly infliximab were the main medications prescribed as the first treatment option. Most participants did not choose ustekinumab, vedolizumab, and certolizumab as their first option. In UC, infliximab, as in CD, was the most prescribed biological therapy. Regarding the biological therapy used as a second option, in the treatment of CD, ustekinumab deserves to be highlighted, as well as vedolizumab in the treatment of UC. The questionnaire also addressed the item of continuing education and the participants responded that the following topics should be addressed and discussed at scientific events: failure of biological therapy was the most requested topic, with 225 responses (80.9%), followed by new drugs with 186 (66.9%) and postoperative follow-up and real-life data, both with 145 (52.2%). 

 In addition to the descriptive analysis of aspects related to the professionals’ performance, the association between time since graduation and specialty, workplace, difficulty in accessing medications, difficulty in referring to a specialist, difficulty in accessing complementary exams and safety in biological therapy with the HDI of the states (very high/high×medium), and the medical ratio based on the cutoff established by the OECD was evaluated. 

 Regarding the relationship between the HDI and the workplace, we observed that private clinics specialized in the treatment of IBDs are more prevalent in places with a medium HDI (p=0.004, p<0.05). Difficulty in accessing medications was greater in locations with a medium HDI for access to methotrexate (p=0.000) in CD and corticosteroids (p=0.015, p<0.05) and 6-mercaptopurine (p=0.009) in ulcerative colitis (UC). Access to the nursing team was the most difficult in places with medium HDI (p=0.016, p<0.05), as was the difficulty in performing double-balloon enteroscopy (p=0.048, p<0.05). 

 We did not find statistically significant results regarding the association of HDI with safety in biological therapy (p=0.884, p>0.05) or correlation with time since graduation (Pearson’s correlation=0.963) and specialty (Pearson’s correlation=0.787). 

 Considering the same qualitative variables, we evaluated the medical workforce. In relation to the workplace, in places that have a medical ratio lower than the cutoff point established by the OECD, there are more doctors working in private hospitals (p=0.002, p<0.05) and public outpatient clinics (p=0.013, p<0.05), than in places with a medical ratio equal to or above the cutoff point established by the OECD. Only in the UC did we observe difficulty in accessing medications, i.e., in places with a medical ratio above or equal to the cutoff point established by the OECD, the greatest difficulty in accessing medications was with azathioprine (p=0.011, p<0.05), 6-mercaptopurine (p=0.045, p<0.05), while biological therapy (p=0.000, p<0.05) was presented as more difficult to access in places with a medical ratio below the cutoff point established by the OECD. 

 Unlike the HDI, in the medical ratio, we did not find significant results regarding the difficulty in referring to a specialist; however, in relation to the difficulty in accessing complementary exams, fecal markers (p=0.023, p<0.05), enterotomography (p=0.03), colonoscopy (p=0.009, p<0.05), capsule endoscopy (p=0.009, p<0.05), and double-balloon enteroscopy (p=0.001, p<0.05) were the exams that were most difficult to access in places with the medical ratio below the cutoff point established by the OECD. We did not find significant results regarding the association between medical reason and safety in biological therapy (p=0.541, p>0.05); however, we observed a very weak correlation with time since graduation and specialty (Pearson’s correlation=0.000), as well as with HDI (Pearson’s correlation=0.022). 

## DISCUSSION

 This study outlined the profile of physicians who treat IBDs, as well as the characteristics of their patients and their greatest difficulties, and also evaluated possible associations be tween care and indices used as measures of development. 

 As it has been observed in research on medical density in Brazil, this study showed a greater number of responses from regions with a higher HDI (or developed) from professionals who, for the most part, work in at least two different places, probably due to low salaries and the high regionalization of professionals, leading to professional, physical, and emotional exhaustion, which can interfere with the quality of care for these complex diseases^14^. 

 In addition to these problems, other difficulties that go beyond medical knowledge and expand and/or are related to public health problems were also analyzed in this study, such as the difficulty in accessing medications. Although most of these medications are distributed by the Unified Health System (SUS), all medications mentioned in the research had some degree of difficulty pointed out, with biological therapy in first place in CD, with 70% of participants, and in UC with 95% of participants. This is due to the delay in updating the Clinical Protocol and Therapeutic Guidelines in UC and CD. In the last update for UC, two biological medications were incorporated (infliximab and vedolizumab), whereas previously there were none; while, for CD, only anti-TNFs were approved for treatment in the collection of these data. The difficulty in accessing these medications is worrying, as they are specific medications not only for the treatment of IBD but also for other diseases, especially immune-mediated^1-3^. 

 Another measure used to assess the quality of care was access to other specialists and non-medical professionals, who assist in the care of these patients. For better treatment of this pathology, it is important to have a multidisciplinary team, and non-medical professionals specialized in this treatment are among the most difficult to refer and monitor together, especially nutritionists, followed by psychologists and nurses, a situation that can be explained by the fact that the multidisciplinary residency was created almost 30 years after the medical residency in the country. 

 Regarding difficulty in access to highly complex exams, the main ones pointed out were double-balloon enteroscopy and capsule endoscopy, followed by calprotectin, entero resonance, and enterotomography. These results are expected, as they are exams with high costs and complexity, available only in some referral centers. Although the number of patients treated by specialized professionals was between 11 and 50 patients with CD and UC, respectively, it is believed that these rates will increase with an increase in epidemiological indices. Considering this fact, we also asked how many patients were being treated with each class of drugs in CD and UC. In CD, we observed that the number of patients using azathioprine and biological therapy was high, while in the treatment of UC, the main medication prescribed was 5-ASA derivatives. Even with the difficulty in accessing some of these medications, the use of prescriptions for these medications is a relevant choice, as they are the main medications that impact the natural history of the disease. 

 Regarding the number of patients using each class of drugs in biological therapy as the first and second medications prescribed, respectively: in CD, infliximab was the first option, followed by ustekinumab; in UC, infliximab was again the most prescribed drug followed by vedolizumab. We can infer that this choice is due to the fact that these medications are the oldest, with greater experience and safety on the part of doctors. 

 In order to detect possible differences in the care provided based on distinct characteristics stratified by the HDI and the OECD average medical index, some variables were analyzed separately. In locations with a medium HDI, there is a greater number of doctors working in private clinics specializing in IBDs, which may indicate a difference in access to healthcare regarding the diagnosis and treatment of IBDs in these states, revealing a disparity in relation to the socioeconomic level for the treatment of this condition, regional differences, and difficulties. Locations with a medium HDI also have greater difficulty in accessing medications, as was observed for CD (methotrexate) and UC (corticosteroids and 6-mercaptopurine). Nursing professionals also have more difficulty accessing these places with a medium HDI. The medium HDI also had an impact on access to double-balloon enteroscopy, which shows us, again, a deficiency related to the health system. 

 When the assessment was made based on the parameter used in relation to the same variables, it was observed that, in relation to the place of work, doctors who live in places where the cutoff point is below the value used by the OECD, a higher proportion of doctors were found working in private hospitals and public outpatient clinics. This may be another indication that, due to the salary deficit of doctors, there is a need for them to have several places of work, regardless of their region or medical reasons. For locations with a medical ratio equal to or greater than 3.5 doctors/1,000 inhabitants, the proportion of doctors who reported difficulty in accessing immunosuppressants such as azathioprine and 6-mercaptopurine was higher, while in locations with a lower medical ratio, doctors had more difficulty in accessing biological therapy. These results indicate that the difficulty of access is possibly not related to the medical workforce, but to other issues, such as the HDI. 

 In regions where medical reasons are lower, access to fecal markers, computed tomography enterography, colonoscopy, capsule endoscopy, and double-balloon enteroscopy was also more difficult. Interestingly, with regard to the difficulty in accessing complementary exams, the medical reason was more decisive than the HDI, with more significant differences, possibly because the need for specialized doctors to carry out these exams is directly related to the medical reason in the population. 

## CONCLUSIONS

 The study showed a portrait of the current profile of doctors who treat IBDs and who participated in this GEDIIB survey. In addition to analyzing and describing the profile of the doctor and his/her difficulties, it listed the main aspects that hinder both diagnosis and treatment, attributed to external factors, independent of his/her reality and competence. These results suggest and demonstrate that for adequate care of patients with IBDs, several external variables beyond medical knowledge and capacity are necessary. Therefore, more effective public health policies should be planned and expanded, with special attention to the rates and results mentioned above, aiming at the growth and adaptation of health systems focused on IBDs. 

## Data Availability

The datasets generated and/or analyzed during the current study are available from the corresponding author upon reasonable request.
